# Supplementary material for: MetaRibo-Seq measures translation in microbiomes
Source: Nat Commun. 2020 Jun 29;11:3268. doi: 10.1038/s41467-020-17081-z (PMC7324362; doi:10.1038/s41467-020-17081-z)
Supplement: Supplementary file 10 — Supplementary Data 7 [file 41467_2020_17081_MOESM10_ESM.zip › File2/Confidence_High_Taxonomy/360582_out.krona.html]

Javascript must be enabled to view this page.

members
magnitude
magnitudeUnassigned
count
unassigned
taxon
rank

360582\_out

3

superkingdom
2
3

3
phylum
976

200643
class
3

3
order
171549

3
family
815

816
genus
3

1

SRS148511\_contig\_number\_18006
species
329854

species
820

SRS018936\_contig\_number\_8607SRS148721\_contig\_number\_32606
2
